# Tetracycline Removal by Activating Persulfate with Diatomite Loading of Fe and Ce

**DOI:** 10.3390/molecules25235531

**Published:** 2020-11-25

**Authors:** Chongning Lv, Jindou Shi, Qiuju Tang, Qi Hu

**Affiliations:** 1 School of Traditional Chinese Materia Medica, Shenyang Pharmaceutical University, Shenyang 110016, China; lcnmi@syphu.edu.cn; 2 School of Pharmaceutical Engineering, Shenyang Pharmaceutical University, Shenyang 110016, China; shijindou1996@foxmail.com (J.S.); tangqiuju96@foxmail.com (Q.T.)

**Keywords:** tetracycline, persulfate, advance oxidation, diatomite, wastewater

## Abstract

Persulfate (PS)-based oxidation technology is efficient in removing refractory organics from water. A novel diatomite (DIA) support Fe and Ce composite (Fe-Ce/DIA) was prepared for activating persulfate to degrade tetracycline in water. The Fe and Ce were uniformly loaded on DIA, and the total pore size of Fe-Ce/DIA was 6.99 × 10^−2^ cm^3^/g, and the average pore size was 12.06 nm. Fe-Ce/DIA presented a good catalytic activity and 80% tetracycline was removed under the persulfate system. The Fe-Ce/DIA also had photocatalytic activity, and the corresponding tetracycline removal efficiency was 86% under UV irradiation. Fe-Ce/DIA exhibited less iron dissolution rate compared with Fe-DIA. The tetracycline degradation rate was enhanced when the temperature increased. The optimal tetracycline removal efficiency was obtained when the conditions were of persulfate 10 mM, Fe-Ce/DIA dosage 0.02 g/L, and tetracycline concentration 50 mg/L. In addition, Fe-Ce/DIA showed a wide pH application and good reusability and stability.

## 1. Introduction

Nowadays, the global pharmaceutical industry develops rapidly, and hundreds of antibiotics have been discovered and applied in human and animal medicine. China consumed 1.62 × 10^5^ tonnes of antibiotics in 2013, and approximately 52% of the antibiotics were used in livestock breeding and aquaculture [1], and the total production increased to 1.96 × 10^5^ tonnes in 2019. Whenever antibiotics are used, wastes with residual antibiotics are inevitable and eventually discharged into the water environment. The concentration of antibiotics in surface water was 10, 15, 50, and 450 µg/L in Europe, the United States, Africa, and the Asia Pacific [2], which may endanger the quality of the aquatic ecological environment [3,4]. Tetracycline (Figure S1), as a representative antibiotic, is widely used in animal production worldwide [5]. China is the biggest supplier of the Active Pharmaceutical Ingredient (API) of tetracycline. The abuse of tetracycline had caused the universal presence of its residuals and metabolites in environments, in particular, these antibiotics are detected by the Wastewater Treatment Plants (WWTPs) [6,7,8,9]. Antibiotics, even in little concentrations, could become a serious threat to ecological security. Thus, effective antibiotic removal approaches should be explored.

Numerous approaches have been employed to remove antibiotics from wastewaters, including biodegradation [10], coagulation/flocculation [11], adsorption [12,13], membrane separation [14], and advanced oxidation processes (AOP) [15,16,17,18]. However, biodegradation methods have difficulty in obtaining suitable microorganisms from those that survive in antibiotics [19,20]; adsorption and membrane methods only accumulate and transfer antibiotics from one to another [21,22,23,24]. Compared with the above, advanced oxidation processes (AOP) can degrade antibiotics by the production of reactive free radicals, such as hydroxyl radical (HO^●^) or sulfate radical (SO_4_^●−^) [25,26]. Fenton is a common AOP process, and H_2_O_2_ is catalyzed by Fe^2+^ to produce HO^●^ for degrading organic pollutants into small molecules such as CO_2_, H_2_O and inorganic salts in the process. However, Fenton is limited by the narrow pH-scale [27,28]. As a Fenton-like reagent, persulfate can be used as the oxidizing agent to produce SO_4_^●−^ to degrade organics in water [29]. SO_4_^●−^ has get increasing attention because of its wider pH range [30], longer half-time, and stronger oxidation selectivity in wastewater treatment [31]. The cost for chemicals and reactors was approximately 0.15–0.75 $/tonne. SO_4_^●−^ can be formed by persulfate and several activators, such as UV-light, heat, base, and electrode transition metal ions [32,33].

Fe is a cheap and abundant crust mineral that is commonly used as a catalyst [34]. The advantages of Fe-based catalysts are as follows: Rich in the earth, easy to produce, and nearly non-toxic [35]. Xu et al. [36] used ferrous ion activating PS to degrade dye orange G, and the decolorization rate of orange G reached 99%. However, the Fe^3+^, formed by the oxidation of Fe^2+^, was easily precipitated into iron mud. The serious iron ion dissolution condition restricts the large-scale application of iron-based catalysts. Therefore, some studies attempted to improve the activity and stability of the catalysts, such as, (1) introduce other metal to improve the catalytic activity; (2) provide a supporter for the catalyst to change the physical structure of the catalyst. Among many metal elements, rare earth is widely used in heterogeneous catalysis for its special redox properties. Cerium (Ce) is one of the most abundant rare earth elements. As a dispersant and stabilizer, it was used to strengthen adsorption and catalytic oxidation effects. The cycle from Ce^3+^ to Ce^4+^ makes CeO_2_ have good electron transfer characteristics and catalytic oxidation activity. The doping of CeO_2_ can improve the storage and activity of lattice oxygen. Perez-Alonso and coworkers reported that the cerium link with iron could increase the performance of catalysts and reduce the dissolution rate of iron [37].

Metal catalysts tend to agglomerate [38,39], and a carrier can help prevent agglomeration [40]. In terms of Fe-based catalysts carrier selection, the current research focuses on new functional materials, such as metal-organic frameworks, carbon nanotubes, graphene oxide, etc., but the complex preparation process and the high price of these materials cause a cost inefficiency. Diatomite (DIA) is porous and cheap, and has strong adsorption capacity [41] and stable chemical properties [42,43]. Some studies used DIA as a carrier: V-doped TiO_2_/DIA showed excellent performance as a photocatalyst for degrading rhodamine [44]; Cu_2_O-ZnO/DIA showed good performance in degrading red water [45]; Ce-doped TiO_2_/DIA granular composite could enhance photocatalytic activity [46]. As is seen from the above, DIA is a potential Fe-based catalysts carrier.

To combine the advantages of Fe/Ce and DIA, a series of DIA matrix composites (Fe-DIA, Ce-DIA, Fe-Ce/DIA) were fabricated to activate persulfate to degrade tetracycline in water. The composites were characterized to show the combination of Fe and Ce. The adsorptive and catalytic ability of DIA containing Fe and Ce was compared through tetracycline removal. The iron dissolution and reusability of the Fe-Ce/DIA were measured. Finally, the parameters for tetracycline removal were given through investigating the Fe-Ce/DIA dosage, tetracycline initial concentration, pH, and temperature in the reaction.

## 2. Results

### 2.1. Characterization of the Composites

**Figure 1:**
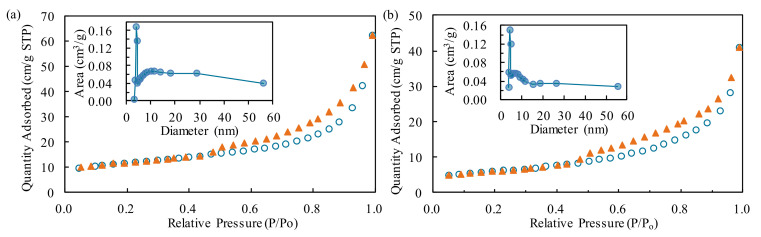
N_2_ adsorption-desorption isotherms of diatomite (DIA) (**a**) Fe-Ce/DIA (**b**); inset: The pore size distribution.

**Figure 2:**
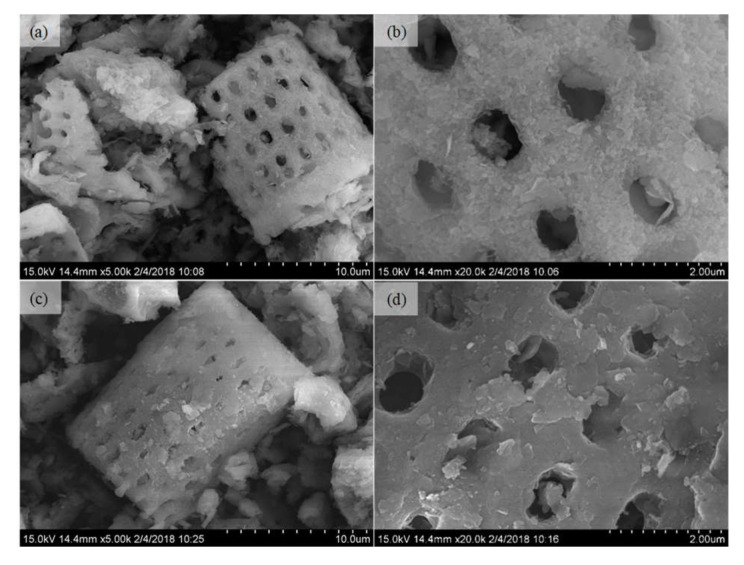
SEM images of DIA (**a**,**b**) and Fe-Ce/DIA (**c**,**d**).

Specific surface areas of the DIA and Fe-Ce/DIA, as determined by the Brunauer–Emmett–Teller (BET) method using N_2_ adsorption-desorption curves, were found to be 40.11 m^2^/g and 21.04 m^2^/g, respectively. The total pore size of DIA and Fe-Ce/DIA was 9.67 × 10^−2^ and 6.99 × 10^−2^ cm^3^/g, and the corresponding average pore size was 9.64 and 12.06 nm (Figure 1), respectively. The surface area and total pore volume decreased with the load of Fe and Ce, but the average pore diameter increased in the process. Some microspores in DIA were blocked by the carried metal and larger diameter holes remained in the process, which decreased the total pore size and increased the average pore diameter. The change might be beneficial for pollutants adsorption based on the previous report [47]. The SEM image indicated that the surface of Fe-Ce/DIA was more compact and the pores were less clear than that of DIA (Figure 2).

**Figure 3:**
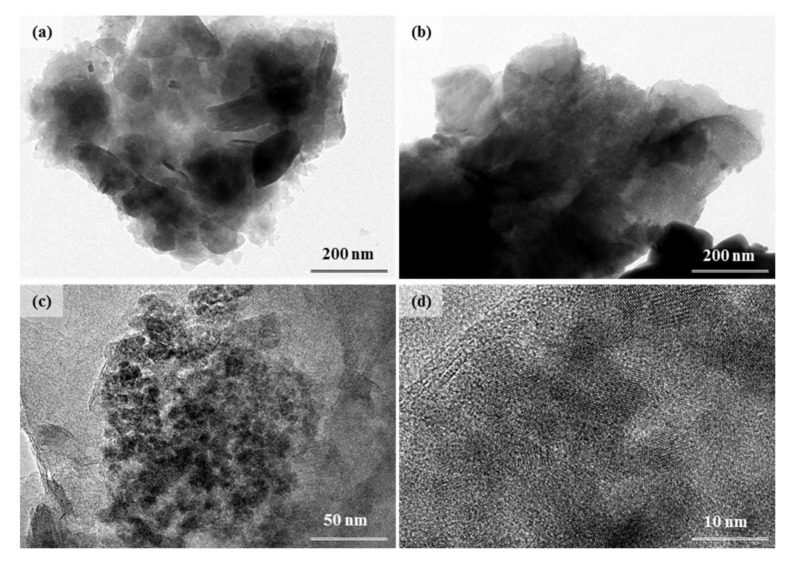
TEM images of DIA (**a**) and Fe-Ce/DIA (**b**–**d**).

To clearly show the microstructure of DIA and Fe-Ce/DIA, Transmission Electron Microscope (TEM) was used to detect the samples (Figure 3). Fe-Ce/DIA was more compact than DIA from TEM photos at 200 nm (Figure 3a,b). At 50 nm, some dark and light areas were shown (Figure 3c): The dark part was Fe_3_O_4_ and the light was CeO_2_, and both Fe and Ce were dispersed into the framework of Fe-Ce/DIA. High-resolution TEM image showed that lattice fringes of CeO_2_ displayed interplanar spacings in the particles (Figure 3d).

**Figure 4:**
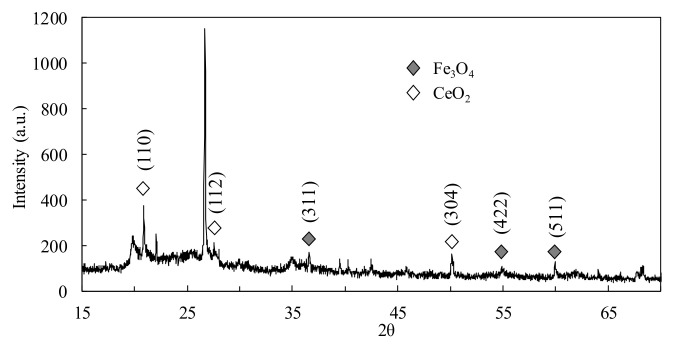
XRD patterns of Fe-Ce/DIA.

Figure 4 depicted the XRD pattern of Fe-Ce/DIA, the diffraction peaks appearing at 36.82°, 55.62°, and 59.30° were attributed to the (311), (422), and (511) diffractive surfaces of the Fe_3_O_4_ apical crystal (Fm-3 m (227), JCPDS no.74-0748), respectively. The peaks at 21.14°, 27.33°, and 51.69° represented (110), (112), and (304) diffractive surfaces of the CeO_2_ fluorite (Fm-3 m (225), JCPDS no. 34-0394), which indicated that Fe and Ce were successfully loaded on diatomite.

### 2.2. The Adsorptive and Catalytic Properties

**Figure 5:**
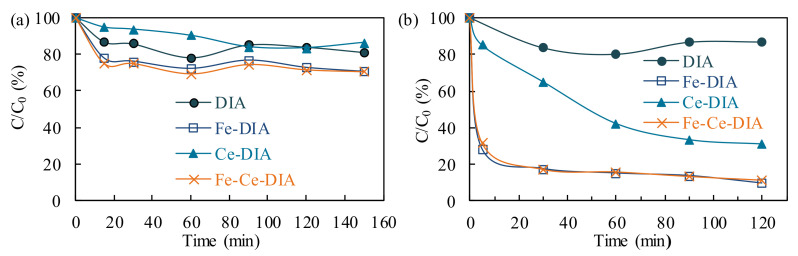
Tetracycline adsorption by DIA, Fe-DIA, Ce-DIA, and Fe-Ce/DIA (**a**); tetracycline degradation by adding persulfate (**b**). Conditions: (Tetracycline) = 200 mL, 50 mg/L, (Catalyst) = 0.02 g, (Persulfate) = 0.134 g, T = 37 °C, 150 rpm.

The tetracycline adsorption by DIA, Fe-Ce/DIA, Fe-DIA, and Ce-DIA was compared under the same conditions (Figure 5). The tetracycline removal by DIA adsorption was about 20% under the dark. The combination of Fe, Ce and DIA could increase the adsorption capacity, and the best efficiency was 30% for Fe-Ce/DIA. The doping of Fe and Ce provided a rough surface, which probably improved the adsorption capacity of Fe-Ce/DIA.

The tetracycline degradation by DIA, Fe-Ce/DIA, Fe-DIA, and Ce-DIA was also investigated through adding persulfate. The load of Fe or Ce could improve the catalytic activity, the removal efficiency by Ce-DIA was about 70%, while that of Fe-DIA and Fe-Ce/DIA were 80% (Figure 5b). The result indicated that Fe-Ce/DIA and Fe-DIA effectively activated persulfate to degrade tetracycline. The Fe-Ce/DIA and Fe-DIA had the same catalytic efficiency in the same dosage, but Fe content in Fe-Ce/DIA was less, which means less iron dissolution.

### 2.3. Photocatalytic Property

**Figure 6:**
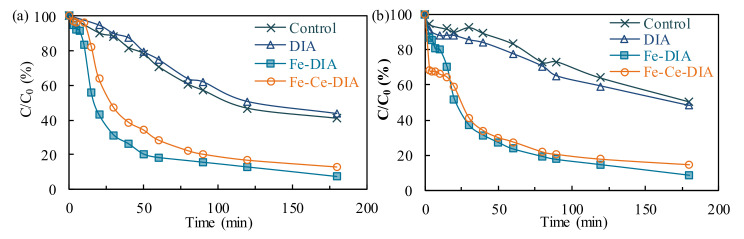
The photocatalytic effect under different light condition: Visible light (**a**); ultraviolet light (**b**).

The photocatalytic activity of Fe-Ce/DIA was investigated under visible and ultraviolet light. The tetracycline removal by Fe-DIA was slightly better than Fe-Ce/DIA, as Fe-DIA contained more Fe in the same dosage (Figure 6a). The final removal rates were 86% (Fe-Ce/DIA) and 91% (Fe-DIA). Besides, CeO_2_ had a strong absorption in the ultraviolet region, and some photogenerated electron pair holes were produced and reacted with water to produce hydroxyl radicals [48,49]. Thus, Fe-Ce/DIA showed better photocatalytic properties than Fe-DIA during the first 20 min (Figure 6b). CeO_2_ exhibited poor photocatalytic activity under visible light, but showed good activity under ultraviolet light (Figure 6). CeO_2_ had a wide band gap of 3.2 eV and only absorbed light with a wavelength less than 444 nm, which led to its little absorption under visible light and thus poor catalytic activity [48].

### 2.4. Metal Leaching and the Reusability of Fe-Ce/DIA

**Figure 7:**
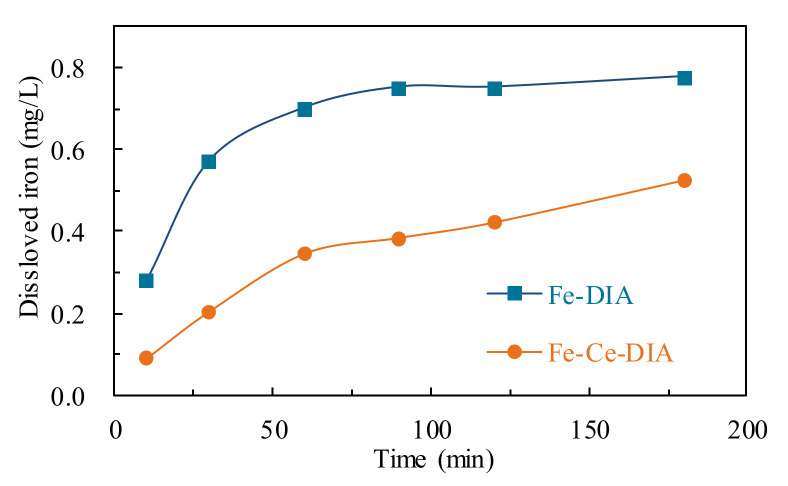
Determination of dissolved Fe in the tetracycline solution.

The dissolved Fe in the catalytic process was detected to show the metal leaching (Figure 7). Dissolved Fe increased rapidly in the first 90 min and then trended to be stable for Fe-DIA, by contrast, the dissolved Fe increased slowly for Fe-Ce/DIA in the process. Ce inhibited the loss of Fe^2+^ and improved the stability of the Fe-Ce/DIA.

**Figure 8:**
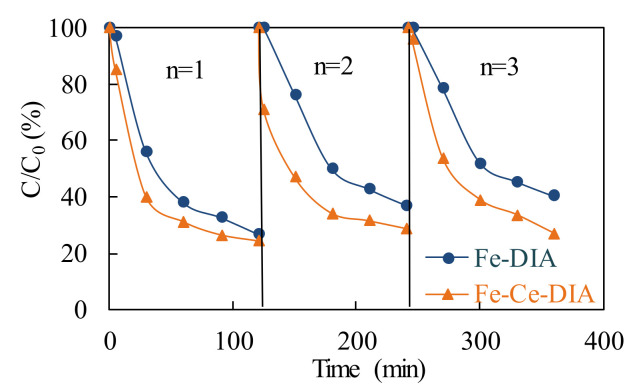
Repeated use of Fe-DIA and Fe-Ce/DIA for TC removal. Conditions: T = 37 °C, 150 rpm.

Reusability is an important factor to evaluate the cost-effectiveness of a catalyst. At the same condition, the tetracycline removal efficiency of Fe-Ce/DIA was better than that of Fe-DIA. Fe-DIA lost the active component (Fe^2+^) and less SO_4_**^−^** was produced during the recycles (Figure 8). After 3 times, Fe-Ce/DIA still showed good catalytic performance.

### 2.5. Parameters on Tetracycline Removal by Fe-Ce/DIA

**Figure 9:**
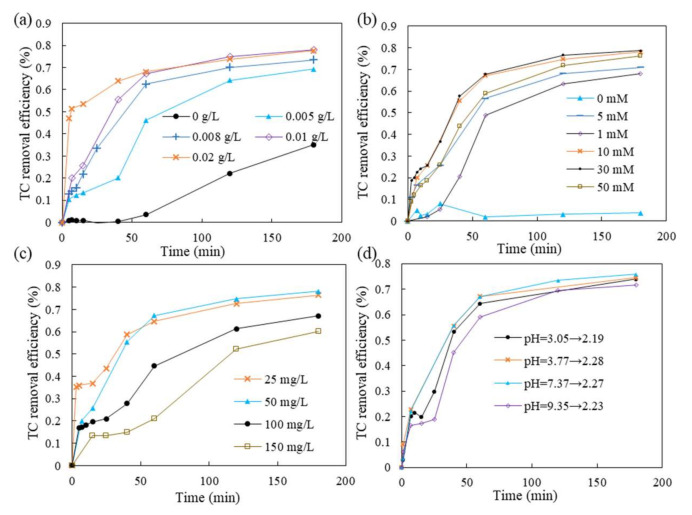
The effects of catalyst dosage (**a**); persulfate dosage (**b**); tetracycline concentration (**c**); pH (**d**) on Tetracycline (TC) removal. Conditions: (Tetracycline) = 200 mL, 50 mg/L, (Catalyst) = 0.02 g, (Persulfate) = 0.476 g, T = 30 °C, 150 rpm.

The effect of Fe-Ce/DIA dosage on the tetracycline removal was investigated at five different dosages. Fe^2+^ was a necessary catalyst for sodium persulfate to generate free radicals. At the same reaction time, higher removal efficiencies were obtained with the dosage from 0 to 0.01 g/L, the reaction rate increased from 2.4 × 10^−3^ min^−1^ to 8.6 × 10^−3^ min^−1^. When the dosage of Fe-Ce/DIA was 0.02 g/L, the reaction rate decreased to 6.1 × 10^−3^ min^−1^ (Figure 9, Table 1). The similar result had been obtained in organic pollutants degradation by activated persulfate [50]. The decline can be explained as follows: Due to the aggregation of excessive catalyst, reactants cannot enter the active center; Fe is consumed by SO_4_^−^ if it exceeds a certain amount as Equation (1) [50]. The optimum dosage of Fe-Ce/DIA was 0.01 g/L in this study. Therefore, considering the cost and efficiency of tetracycline removal, a dosage of 0.01 g/L Fe-Ce/DIA was selected for the successive tetracycline removal study.

SO_4_^−^ + Fe^2+^ → SO_4_^2^^−^ + Fe^3+^ (1)

The dosage of sodium persulfate was an important parameter on tetracycline removal. A higher reaction rate was obtained with a higher sodium persulfate dosage from 1 to 30 mM, but the reaction rate decreased when the dosage of sodium persulfate exceeded 30 mM (Figure 9, Table 2). This phenomenon can be explained by the increase of SO_4_^−^, which is obtained from the increasing dosage of sodium persulfate. Moreover, the collision probability between SO_4_^−^ and tetracycline was increased, and the combined action of the two accelerated the reaction rate, so the reaction rate constantly increased. However, when the dosage of sodium persulfate was increased to a certain extent, as the interaction between SO_4_^−^ and S_2_O_8_^2−^, SO_4_^−^ would react with S_2_O_8_^2−^ to form SO_4_^2−^ and S_2_O_8_^−^· (Equations (2) and (3)). Besides, SO_4_^−^ may inhibit the formation of HO^●^. In this study, 10 mm was selected as a proper dosage for tetracycline removal.

SO_4_^●^^−^ + SO_4_^●^^−^ → S_2_O_8_^2^^−^ (2)

SO_4_^●^^−^ + S_2_O_8_^2^^−^ → SO_4_^2−^ + S_2_O_8_^●−^ (3)

The effect of the initial tetracycline concentrations on tetracycline removal was also investigated. The removal efficiency decreased with an increase in initial concentrations (Figure 9c, Table 3). As the number of producing free radicals was maintained the same, the increase in initial tetracycline concentration led to the reduction of tetracycline oxidized by SO_4_^●^^−^ per unit volume in unit time.

The initial pH was an important parameter on tetracycline removal because it can affect the activation of persulfate and the existent forms of tetracycline (Figure 9d, Table 4). Tetracycline had four forms under different pH conditions (H_3_TC^+^, H_2_TC, HTC^−^, and TC^2^^−^). Fe^2+^ was stable in acid condition and produced more SO_4_^−^, but in the alkaline condition, SO_4_^−^ could be converted into HO^●^, and SO_4_^−^ was quenched by the increasing HO^●^. The results were presented in the Figure 9d and Table 4. It could be observed than the reaction rate was the same under different pH conditions, consistent with Zhao et al. [51]. The decrease of pH value may be due to the small organic acid molecules and HSO_4_^−^·produced in the reaction process. The final pH of the reactions was 2.19–2.33, which were in the same range.

The effect of temperature on tetracycline removal was evaluated at 25, 30, 35, and 40 °C. The tetracycline removal efficiency increased with the temperature increasing (Table 5). As more energy was absorbed in higher temperatures, the O-O bond in sodium persulfate was more likely to break, resulting in more SO_4_^●^^−^.

### 2.6. Degradation Mechanism

**Figure 10:**
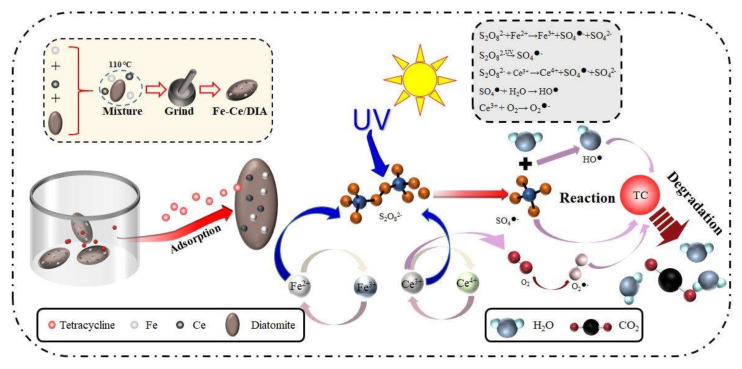
Potential mechanism of activation of persulfate by Fe-Ce/DIA to degrade tetracycline.

Fe-Ce/DIA showed good performance on tetracycline removal, and a potential removal mechanism was proposed as shown in Figure 10. Firstly, Tetracycline and persulfate were absorbed on the surface of Fe-Ce/DIA. Tetracycline antibiotics were positively charged in acidic solutions and anionic under alkaline conditions [52]. DIA was negatively charged at most pH conditions. In acid condition, tetracycline adsorption by diatomite depended on positive and negative charge; in alkaline condition, tetracycline was adsorbed by cation on diatomite. Fe^2+^ could activate persulfate to produce SO_4_^●^^−^. Ce^3+^ can also activate persulfate to produce SO_4_^−^ [28]. The persulfate could be activated by ultraviolet light [8,53]: SO_4_^●^^−^ reacted with H_2_O to form HO^●^ radicals, and Ce^3+^ activated O_2_ to produce O_2_^●−^. Finally, tetracycline was degraded into H_2_O and CO_2_ by all active radicals (HO^●^, SO_4_^●^^−^ and O_2_^●−^). Eight main products were found in the degradation process, and the degradation products included m/z 475, 459, 443, 430, 441, 414, 386, and 342. The detail results were shown in Table S1 and Figure S2.

## 3. Materials and Methods

### 3.1. Chemicals

Diatomite (DIA, CP) was purchased from Ruijinte Chemical Co., Ltd. (Tianjin, China). Barium nitrate hexahydrate (Ce(NO_3_)_2_·6H_2_O) was procured from Guangfu Fine Chemical Research Institute (Tianjin). FeSO_4_, NH_3_·H_2_O, and NaOH were acquired from Damao Chemical Reagent Factory (Tianjin). Sodium chloride, methanol and sulfuric acid were obtained from Keanlong Bohua Pharmaceutical Chemical Co., Ltd. (Tianjin). Citric acid, sodium sulfate, ammonium ferrous sulfate, and hydroxylamine hydrochloride were acquired from Bodi Chemical Co., Ltd. (Tianjin, China). 1, 10-Phenanthroline was purchased from Sinopharm Chemical Reagent Co., Ltd. (Shanghai, China). All chemicals were of analytical grade.

### 3.2. Synthesis of Composites

DIA (3 g) was calcined at 300 °C for 2 h, mixed with NaCl solution, stirred in a water bath at 65 °C for 2 h, washed until no Cl^−^ detection, and dried at 110 °C. The solid-liquid ratio of DIA and NaCl (60 mL 1.0 mol/L) was 1:20.

Ce(NO_3_)_2_·6H_2_O (0.043 g) was dissolved in water (50 mL) and mixed with FeSO_4_ (1.043 g). The total concentration of Fe^2+^ and Ce^2+^ was 0.1 mol/L. The solution was mixed with dried DIA, added with citric acid (1.051 g) and stirred in a water bath (60 °C for 2 h, 80 °C for 1 h). Ammonia water was added during the reaction to maintain the precursor pH = 8.0. The material was dried at 110 °C, calcined at 400 °C for 2 h, and ground to obtain Fe-Ce/DIA. The Fe-DIA and Ce-DIA were also synthesized by the same method as Fe-Ce/DIA.

### 3.3. Characterization of Composites

X-ray diffractometer (XRD, Bruker D8 Advance, Karlsruhe, Germany) was used to test the crystal properties. Scanning electron microscopy (SEM, JEM1002EX) was used to analyze microscopic morphology and element distribution. The pore structure and specific surface area characteristics of the composites were tested using a physical adsorption instrument (BET, ASAP 2460, Micrometrics, Norcross, GA, USA).

### 3.4. Adsorption Study

The adsorption capacity of Fe-Ce/DIA, Fe-DIA, Ce-DIA, and DIA to tetracycline was investigated before a degradation study. Each sample (0.020 g) was dissolved in tetracycline (200 mL, 50 mg/L) separately, then reacted in dark for 2.5 h (37 °C, 150 rpm). The sample (3 mL, reacted for 0, 30, 60, 90, 120, and 180 min) was filtered using a 0.22 μm filter, extracted in EP tube for 2 mL, mixed with CH_3_OH (3 mL), and measured for absorbance at 359 nm. The basic calculations in adsorption can be found in previous study [32,54].

### 3.5. Degradation Study

The tetracycline degradation by Fe-Ce/DIA, Fe-DIA, Ce-DIA, and DIA was investigated. Each sample (0.02 g) was dissolved in tetracycline (200 mL, 50 mg/L), added with sodium persulfate (0.134 g) to react in dark for 2 h (37 °C, 150 rpm). The samples (3 mL, reacted for 0, 30, 60, 90, and 120 min) were processed and measured as described above.

The effect of visible light and ultraviolet on tetracycline degradation were investigated. Each sample (0.02 g), Fe-Ce/DIA, Fe-DIA, Ce-DIA, and DIA, was dissolved in tetracycline (200 mL, 50 mg/L) separately, and then sodium persulfate (0.426 g) was added to the system. The reaction was under Xe lamp (25 A, 500 W) and Hg lamp (5.2 A, 350 W) with magnetic stirring at 650 rpm.

A UPLC system coupled with a Bruker’s Campact high-resolution time-of-flight (TOF) mass spectrometer was applied to the UPLC-MS/MS analysis of samples to identify the structure of the degradation products of tetracycline.

Chromatographic separation was achieved using an C18 column (2.1 mm × 100 mm, 1.7 μm). The column temperature was maintained at 30 °C, and the mobile phase, at a flow rate of 0.2 mL/min, consisted of solvent A (0.1% acetic acid in water) and mobile phase B (methanol). The gradient program of the mobile phase was as follows: 0–20 min, 15–55% B. Mass spectrometry conditions: Electrospray interface (ESI), MS analysis in negative ion mode, mass-nucleus ratio scan range 100–800, collision energy is 12 eV.

### 3.6. Batch Study of Tetracycline Removal

The effect of the various parameters, including the dosage of composites (Fe-Ce/DIA, Fe-DIA, Ce-DIA, and DIA) and sodium persulfate, initial pH and tetracycline concentrations, were investigated in details. The reusability of the composites (Fe-Ce/DIA and Fe-DIA) were performed by placing 0.002 g Fe-Ce/DIA or Fe-DIA in 200 mL of 50 mg/L tetracycline solution and adding sodium persulfate of 0.476 g, respectively.

## 4. Conclusions

A series of composites (Fe-Ce/DIA, Fe-DIA, Ce-DIA) were synthesized to remove tetracycline from water. The average pore size of Fe-Ce/DIA was larger than DIA, which was beneficial to tetracycline adsorption. SEM and TEM images showed Fe and Ce were uniformly dispersed in DIA. The tetracycline removal efficiency was 80% at the catalytic oxidation system provided by Fe-Ce/DIA and persulfate. Ultraviolet light could promote the removal efficiency, and 86% tetracycline was removed by Fe-Ce/DIA activating persulfate under ultraviolet light. Eight main products were found in the tetracycline degradation process. A recommended parameter was of Fe-Ce/DIA dosage 0.02 g, persulfate 10 mM, and initial tetracycline solution 50 mg/L. The iron dissolution of Fe-Ce/DIA was less than Fe-DIA in activating persulfate. Fe-Ce/DIA showed wide pH application and excellent reusability in tetracycline removal. The metal doped diatomite is a promising catalyst in antibiotics pollution control.

## Figures and Tables

**Table 1:** The reaction rate of tetracycline degradation at different dosages of Fe-Ce/DIA.

Dosage of Fe-Ce/DIA (g/L)	Reaction Rate Constant *k* (min^−^^1^)	R^2^
0	2.4 × 10^−^^3^	0.9928
0.005	6.9 × 10^−^^3^	0.9468
0.008	7.5 × 10^−^^3^	0.8700
0.010	8.3 × 10^−^^3^	0.8473
0.020	6.1 × 10^−^^3^	0.7022

**Table 2:** The reaction rate of tetracycline degradation at different dosages of persulfate.

Dosage of PS (mM)	Reaction Rate Constant *k* (min^−^^1^)	R^2^
0	0.6 × 10^−^^4^	0.0782
1	6.7 × 10^−^^3^	0.9080
5	7.1 × 10^−^^3^	0.9061
10	8.3 × 10^−^^3^	0.9094
30	8.5 × 10^−^^3^	0.9016
50	8.2 × 10^−^^3^	0.9203

**Table 3:** The reaction rate of tetracycline degradation at different initial concentrations.

Concentration of TC (mg/L)	Reaction Rate Constant *k* (min^−^^1^)	R^2^
25	9.2 × 10^−^^3^	0.8321
50	8.3 × 10^−^^3^	0.8473
100	6.0 × 10^−^^3^	0.006
150	5.3 × 10^−^^3^	0.0053

**Table 4:** The reaction rate of tetracycline degradation at different pH.

pH of Solution	Reaction Rate Constant *k* (min^−^^1^)	R^2^
3.05	7.4 × 10^−^^3^	0.8089
3.77	7.3 × 10^−^^3^	0.7661
7.34	8.0 × 10^−^^3^	0.8179
9.53	7.5 × 10^−^^3^	0.8780

**Table 5:** The reaction rate of tetracycline degradation at different temperatures.

T (°C)	Reaction Rate (min^−^^1^)	R^2^
25	1.81 × 10^−^^2^	0.9194
30	1.83 × 10^−^^2^	0.9920
35	1.86 × 10^−^^2^	0.9686
40	1.98 × 10^−^^2^	0.9524

## Supplementary Materials

The following are available online Table S1: The intermediate products of the tetracycline degradation, Figure S1: The structure of tetracycline, Figure S2: The BPC of intermediate products of the tetracycline degradation.
