# Supplementary material for: Tetracycline Removal by Activating Persulfate with Diatomite Loading of Fe and Ce
Source: Molecules. 2020 Nov 25;25(23):5531. doi: 10.3390/molecules25235531 (PMC7728345; doi:10.3390/molecules25235531)
Supplement: Supplementary file 1 [file molecules-25-05531-s001.pdf]

Supporting Material

Article

Tetracycline removal by activating persulfate with diatomite loading of Fe and Ce

Chongning Lv[1], Jindou Shi[2], Qiuju Tang[2], Qi Hu[2, *]

[1] School of Traditional Chinese Materia Medica, Shenyang Pharmaceutical University, Shenyang 110016, P.R. China

[2] School of Pharmaceutical Engineering, Shenyang Pharmaceutical University, Shenyang, Shenyang 110016, P.R. China

* Correspondence: huqi@syphu.edu.cn; Tel.: +86-24-43520205

Summary

Table S1. The intermediate products of the tetracycline degradation

Figure S1. The structure of tetracycline

Figure S2. The BPC of intermediate products of the tetracycline degradation

Table S1. The intermediate products of the tetracycline degradation.

m/z	Retention times（min）	Chemical structure	First order spectrum
459.1296	4.76	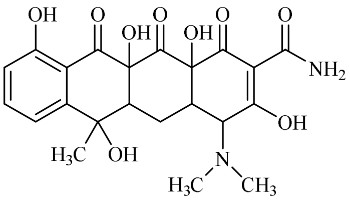	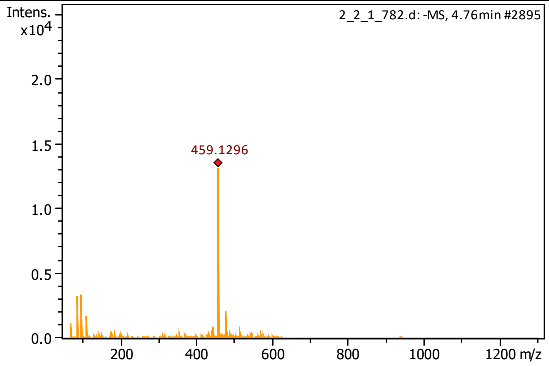
475.1244	5.15	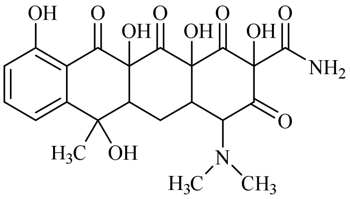	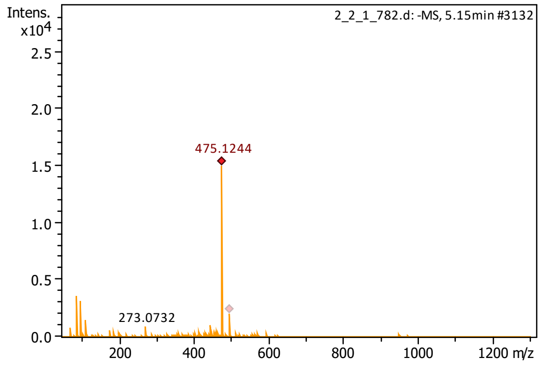
443.1347	6.84	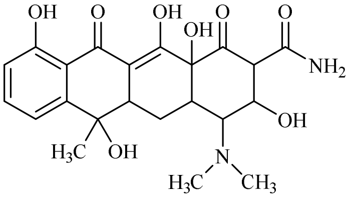	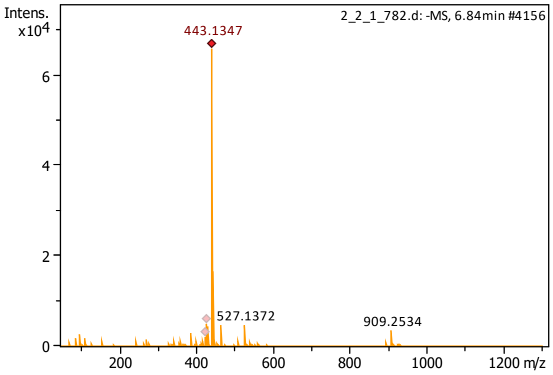
430.0641	7.91	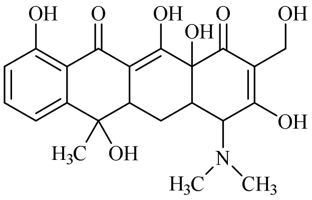	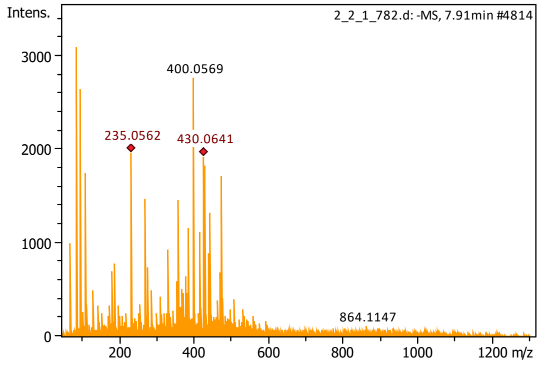

441.1181 13.49

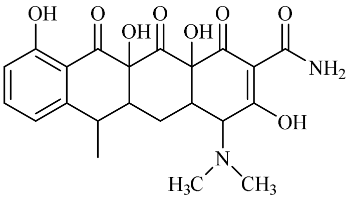

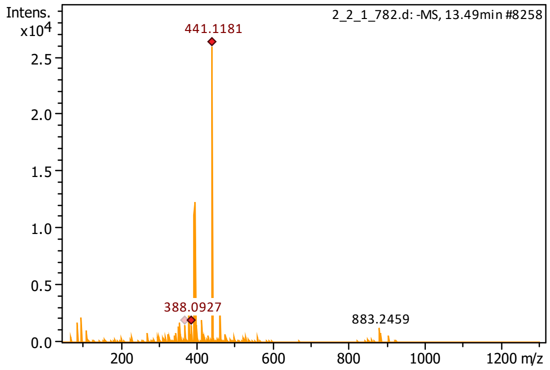

386.0734 14.51

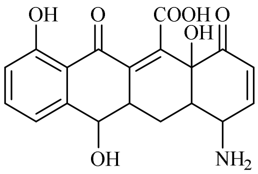

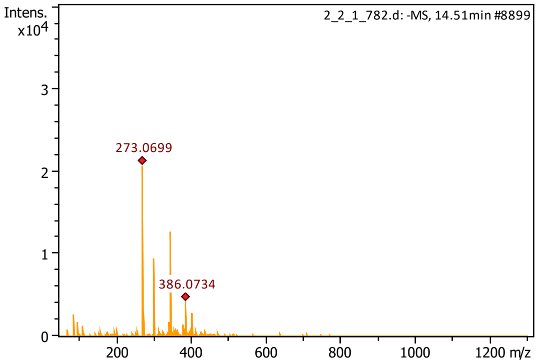

414.0717 15.01

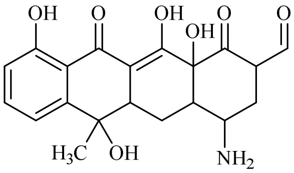

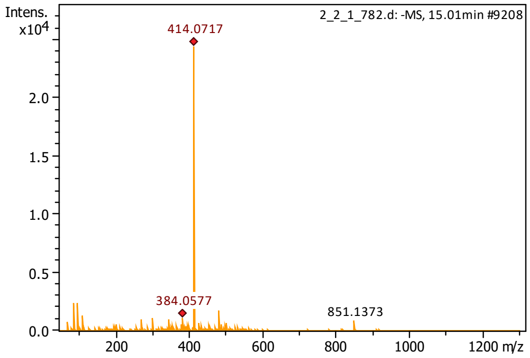

342.1266 16.07

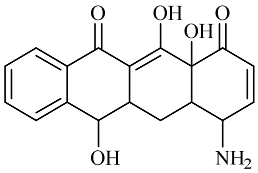

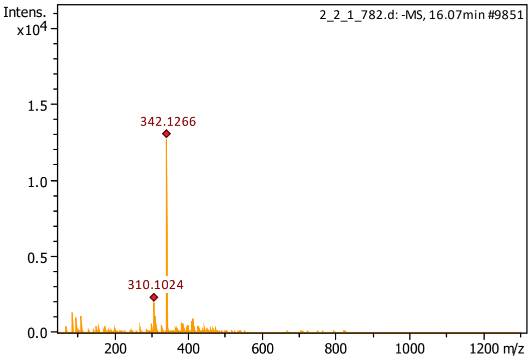

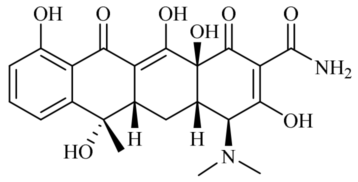

Figure S1. The structure of tetracycline.

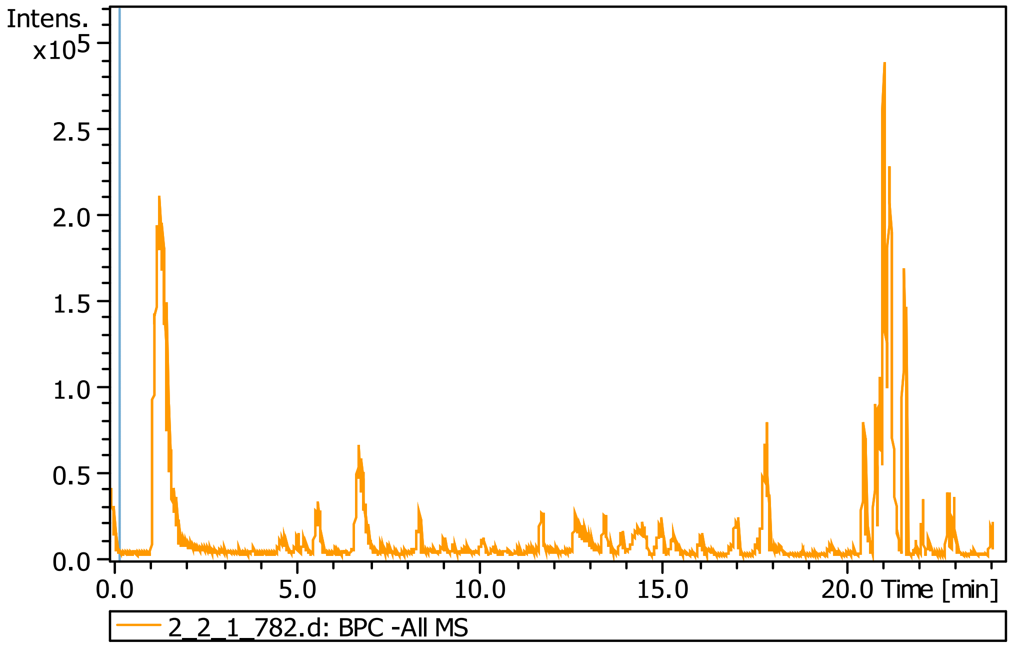

Figure S2. The BPC of intermediate products of the tetracycline degradation.
